# ATQ: alert time quality, an evaluation metric for assessing timely epidemic detection models within a school absenteeism-based surveillance system

**DOI:** 10.1186/s12889-023-15747-z

**Published:** 2023-05-10

**Authors:** Kayla R. Vanderkruk, Lorna E. Deeth, Zeny Feng, Lise A. Trotz-Williams

**Affiliations:** 1 Department of Mathematics and Statistics, University of Guelph, Stone Road, N1G 2W1 Guelph, Canada; 2 Wellington-Dufferin-Guelph Public Health, Chancellors Way, N1G 0E1 Guelph, Canada

**Keywords:** Absenteeism surveillance system, Influenza, Epidemic detection, Simulation study, Evaluation metric

## Abstract

**Background:**

Wellington-Dufferin-Guelph Public Health (WDGPH) has conducted an absenteeism-based influenza surveillance program in the WDG region of Ontario, Canada since 2008, using a 10% absenteeism threshold to raise an alert for the implementation of mitigating measures. A recent study indicated that model-based alternatives, such as distributed lag seasonal logistic regression models, provided improved alerts for detecting an upcoming epidemic. However model evaluation and selection was primarily based on alert accuracy, measured by the false alert rate (FAR), and failed to optimize timeliness. Here, a new metric that simultaneously evaluates epidemic alert accuracy and timeliness is proposed. The alert time quality (ATQ) metric is investigated as a model selection criterion on both a simulated and real data set.

**Methods:**

The ATQ assessed alerts on a gradient, where alerts raised incrementally before or after an optimal day were considered informative, but were penalized for lack of timeliness. Summary statistics of ATQ, average alert time quality (AATQ) and first alert time quality (FATQ), were used for model evaluation and selection. Alerts raised by ATQ and FAR selected models were compared. Daily elementary school absenteeism and laboratory-confirmed influenza case data collected by WDGPH were used for demonstration and evaluation of the proposed metric. A simulation study that mimicked the WDG population and influenza demographics was conducted for further evaluation of the proposed metric.

**Results:**

The FATQ-selected model raised acceptable first alerts most frequently, while the AATQ-selected model raised first alerts within the ideal range most frequently.

**Conclusions:**

Models selected by either FATQ or AATQ would more effectively predict community influenza activity with the local community than those selected by FAR.

**Supplementary Information:**

The online version contains supplementary material available at 10.1186/s12889-023-15747-z.

## Introduction

Seasonal influenza epidemics cause significant morbidity and mortality each year [1], with the duration and severity of influenza epidemics varying year-to-year. Early epidemic detection at the regional level can be conducive in reducing the impact of influenza by prompting public health authorities to implement mitigating strategies. Syndromic surveillance methods focus on behaviours that occur due to symptoms, such as non-prescription drug sales [2, 3], absenteeism from work or school, and web queries [4, 5]. In spite of often being non-specific to community illness, these methods tend to have a level of sensitivity that can allow them to identify disease outbreaks, or the start of a seasonal epidemic, earlier than typical surveillance methods that monitor laboratory-reported cases (which are often lagged) [6, 7], thereby making it possible for public health agencies to respond quickly.

On March 11, 2020, COVID-19 was announced as a pandemic by the World Health Organization (WHO). With the availability of vaccines, and with individuals recovering from infections, populations of some countries and regions are moving toward “herd resistance", where infections continue but the protection from vaccinations and prior infections help prevent infected people from severe illness or death. COVID-19 is thought likely to become seasonal like influenza, and no longer pose a tremendous burden on the health care system or cause disruptions to society on the large scale seen during the first two years of pandemic [8]. In this case, COVID-19 would follow the footprint of seasonal influenza, and methodology development on surveillance of influenza epidemics as we propose in this paper would shed light on the strategy of surveillance of the COVID-19 epidemic and/or mixed epidemics of different infectious diseases.

Children typically have the highest influenza infection rates among all age groups [9]. In general, children are encouraged to stay home when ill, therefore monitoring school absenteeism can provide early signs of community infection [10, 11]. The school absenteeism-based surveillance program has been employed and conducted by Wellington-Dufferin-Guelph Public Health (WDGPH) since 2008. Schools within the Upper Grand District School Board (UGDSB) voluntarily report daily absenteeism using an online form. When student absenteeism surpasses 10% in any given school, an alert is generated in the data system and WDGPH follows up to determine if the high absenteeism is illness related and if so, advise on mitigating measures. School absenteeism surveillance programs in other jurisdictions have used similar absenteeism thresholds [11–14]; however, this approach was found to be ineffective for schools with high baseline absenteeism levels and provide inadequate lead time for public health officials to implement mitigation strategies [12]. Model-based alternatives to the 10% threshold method have recently been studied, and were found to improve epidemic alert accuracy [10]. However, the model selection metrics employed were limited since they optimized alert accuracy using a false alert rate (FAR), but did not necessarily optimize the timeliness of the alerts raised [10]. Alerts that are accurate but not timely may provide insufficient time for public health intervention. This paper proposes a novel metric, alert time quality (ATQ), to evaluate alerts raised by a school absenteeism surveillance prediction model in terms of both accuracy and timeliness. Summary statistics of the ATQ serve as a criterion for model selection and tuning parameter selections when multiple statistical models with their associated turning parameters are considered for raising an epidemic alert. To assess the ATQ metric, school absenteeism and influenza data from WDGPH are used to fit models and compare alerts raised when using the ATQ versus the FAR metric proposed by Ward *et al.* (2019) [10].

Furthermore, as there is no available method or software to simulate school absenteeism and influenza confirmed case data, in this paper we developed and assembled a novel simulation procedure to generate these data. This data simulation procedure can be further adapted to generate school absenteeism data in other regions and confirmed case data for other seasonal infectious diseases. For example, as COVID-19 might become seasonal and now, official confirmed cases in Canada are only relying on self reported cases and positive results from PCR tests which are only available to vulnerable groups, our simulation model can be adapted to simulate COVID-19 confirmed cases. As schools were closed multiple times for varying durations during the COVID-19 pandemic (schools in different provinces and regions across Canada had been closed several times during March 2020 to January 2022 due to the surge of the COVID-19 infections), it was impossible to collect absenteeism data with acceptable quality. To address this gap, our developed simulation procedure can be adapted to generate data to facilitate research in this area. In this paper, our proposed surveillance methods will be further evaluated using the data generated by our simulation model that mimics absenteeism and influenza data observed in the WDG community.

This paper is organized as follows: description of real WDGPH data, statistical models for epidemic detection, the proposed ATQ and ATQ-based metrics, and our simulation study with the proposed data simulation procedure will be organized in the Methods section. Results from the analysis of real WDGPH data and simulation study, and the alert quality analysis, will be presented in the Results section. We will conclude the paper with discussions and future work.

## Methods

This section includes five parts: (1) a description of WDGPH school absenteeism and influenza data, (2) the statistical models used for epidemic detection, (3) the development of ATQ, (4) model evaluation using ATQ summary statistics, and (5) the simulation model for simulation study.

### Absenteeism and influenza data

Two data sets were provided by WDGPH: (1) absenteeism data for schools within the UGDSB, and (2) laboratory-confirmed influenza cases within WDG. Together, in this study, these data sets formed the basis of the epidemic detection model, where the start of the annual influenza epidemic was defined by the influenza data, and this start was predicted using the absenteeism data.

Influenza and school absenteeism data were available from January 2008 to June 2018. In Canada, a school year runs from early September to late June. Therefore, the partial school year from January to June 2008 was omitted from this analysis. Additionally, the 2009-10 school year was excluded from this study since the H1N1 pandemic occurred during this school year and was therefore not representative of a typical influenza season. Overall the study period included nine complete school years, from September 2008 to June 2018 with the 2009-10 school year excluded. All data analyses and visualizations were performed using R version 3.5 [15].

#### Absenteeism data set

Collection of data from schools with the UGDSB for daily absenteeism surveillance had been done by WDGPH via an online form. The absenteeism data set obtained from WDGPH contained records (one per school per day) with information on school type (elementary or secondary), anonymized school identifier, anonymized school catchment area identifier, the number of students in each school (referred to herein as school size), and the number of absent students each day for participating schools. Daily all-cause absenteeism percentages were calculated by dividing the number of absent students in a school by the school size. Data had not been collected on days students did not have to attend school, such as weekends, school holidays, and professional activity days. Following results from a previous study, these non-school days were removed from the data set [10]. A total of 88 elementary schools and 14 secondary schools had reported to WDGPH during the study period. Because attendance reporting was voluntary, not all schools within the UGDSB participated, and participating schools may not have consistently reported daily absences. In a previous study, models that exclusively used elementary school absenteeism data consistently raised influenza alerts with higher accuracy than those that used secondary school absenteeism either exclusively or in combination with elementary school absenteeism [10]. Therefore, the current analysis used only elementary school absenteeism, and for the remainder of the study, the term “school(s)" refer solely to elementary school(s).

Since absenteeism data had been manually entered by schools, they were prone to data entry errors such as mistypes. For example, a school with a school size of 500 may have accidentally reported 5000 students on a given day. Thus, records where a school reported a population of fewer than 45 or greater than 820 students, the smallest and largest consistently reported school sizes respectively, were excluded from the analysis. Similarly, observations in which a school reported a daily absenteeism of 50% or more were removed for suspicion of data entry error. Additionally, if a school reported absenteeism on fewer than five days over the entire study period, all data for that school were excluded. If a school had submitted duplicate reports on a given day, the maximum absenteeism percent for the day was used. Mean daily all-cause absenteeism percentage was calculated for each day using data from all schools reporting on that day.

#### Influenza data set

The influenza data set provided by WDGPH included all de-identified laboratory-confirmed influenza cases and the date on which each case was reported to the public health unit during the study period. Influenza cases were aggregated by day to provide a count of the confirmed cases, and a binary variable was created to indicate if at least one case had occurred on each day of the study period. For this study, the influenza season was defined as the period from September 1 of each year to August 31 of the following year. For each influenza season, the reference date was defined as the report date of the second of two laboratory-confirmed influenza cases reported within a seven day period of each other for the first time within an influenza season, even if both had been reported on the same day. The reference date was used to indicate the start of the actual seasonal influenza epidemic within the community, as opposed to the sporadic cases that often occur at the beginning of an influenza season that might not necessarily be an indication of the start of the influenza epidemic, the situation of a rapid transmission of the disease within the population.

### Epidemic detection models

The current practice for WDGPH is to follow-up with schools when school absenteeism surpasses 10% in any given school, to determine whether the high absenteeism is related to increased transmission of communicable diseases (including influenza) within that school. Since this study focused on region-wide alerts, aggregated, rather that school level, absenteeism data were used to raise a region-wide epidemic alert when the mean daily absenteeism surpassed 10%. This 10% threshold method was compared to model-based alternatives, which have been investigated as a potential improvement upon the current 10% threshold method [10]. In particular, in that work, distributed lag logistic regression models with fixed effects for seasonality and a random effect for school year consistently outperformed other models [10], lending itself to be the primary model type of interest.

Following the notation of Ward *et al.* (2019) [10], the region-wide seasonal mixed effects logistic regression model was given by:

1 $$\begin{aligned} \text {logit}(\theta _{tj}) =&\beta _0 + \beta _1 x_{tj} + \beta _2 x_{(t-1)j} + ... + \beta _{l+1} x_{(t-l)j}\nonumber \\&+ \beta _{l+2} \sin _j(\frac{2\pi t^*}{T^*}) + \beta _{l+3} \cos _j(\frac{2\pi t^*}{T^*}) + \gamma _{j}, \end{aligned}$$

where the outcome of interest, $\theta _{tj}$, was the probability of at least one case being reported on day *t* in school year *j*. The main predictor variables, $x_{(t-i)j}$, were the lagged mean daily absenteeism percentages, where lag time was from 0 up to *l*. The trigonometric functions captured the seasonal effect of influenza, where $t^*$ represented the calendar day of the year on which $x_{tj}$ was observed, and $T^*=365.25$. The random effect for the *j*th school year, $\gamma _j$, was assumed to follow a $N(0,\tau ^2)$ distribution and accounted for intracorrelation among daily absenteeism and influenza observations within a given school year, but varied over different school years.

Additional models were explored to include indicator covariates for the day of the week (DOW), Monday through Friday, to account for their effects on absenteeism. For all versions of the seasonal mixed effects models, lag values $l=1, \dots ,15$ were considered. The glmr function from the lme4 package in R was used to fit the aforementioned models [15, 16].

Data from the first full school year available were used for training, and for each subsequent school year the model was trained on data from all prior years and September of the current year. Refitting the model annually allows for improvements in model covariate estimates as more data become available. Data from October to the reference date of the year of interest were used to raise epidemic alerts and evaluate model performance. Days with no absenteeism data reported, including weekends, were considered as missing values and therefore were removed from the analysis [10].

### Alert evaluation metrics

A model was considered to generate an alert on day *t* of school year *j* if the predicted probability of at least one laboratory-confirmed influenza case, $\theta _{tj}$, was greater than a threshold $\theta$. Threshold values between 0.10 and 0.60, in increments of 0.05, were considered. The ATQ-based and FAR metrics were used to select an optimal threshold, $\theta ^*$, and lag value, *l*. Any alerts raised after the reference date and before the beginning of the following school year were ignored.

The study by Ward *et al.* (2019) proposed two metrics, FAR (referred to as the false *alarm* rate) and accumulated days delayed (ADD), to evaluate the respective accuracy and timeliness of alerts raised by an epidemic prediction model [10]. In that study, true alerts were defined as alerts that are raised within the 15 calendar day period prior to and including the reference date, while alerts raised prior to this period are considered false alerts [10]. Readers are referred to Ward *et al.* (2019) for details of these evaluation metrics, but in brief the FAR for year *j* was defined as:

2 $$\begin{aligned} FAR_j = \left\{ \begin{array}{ll} \frac{n_f}{n_f +1} &{} \text {if a true alert was raised} \\ 1 &{} \text {if no true alerts were raised,}\\ \end{array}\right. \end{aligned}$$

where $n_f$ was the number of false alerts raised in school year *j* [10]. Models that produced the smallest average FAR and ADD values were favoured, however simultaneously optimizing two separate metrics was shown to be difficult. Consequently, Ward *et al.* (2019) prioritized the model that had the minimum FAR, and the ADD served as a tiebreaker [10]. In Ward *et al.* (2019) true alerts were strictly defined as alerts raised between the reference date and 14 days prior, and the optimal alert was exactly 14 days prior to the reference date [10]. An alert raised even one day before the optimal alert day was considered to be a false alert, which makes the FAR and ADD metrics appear too rigid in defining a true alert. These limitations motivated the development of a metric which can account for both accuracy and timeless of raised alerts. In this paper, we propose a novel metric, alert time quality (ATQ), to provide a gradient approach to optimizing epidemic prediction models based on alert timeliness and accuracy.

#### Alert time quality

**Fig. 1:**
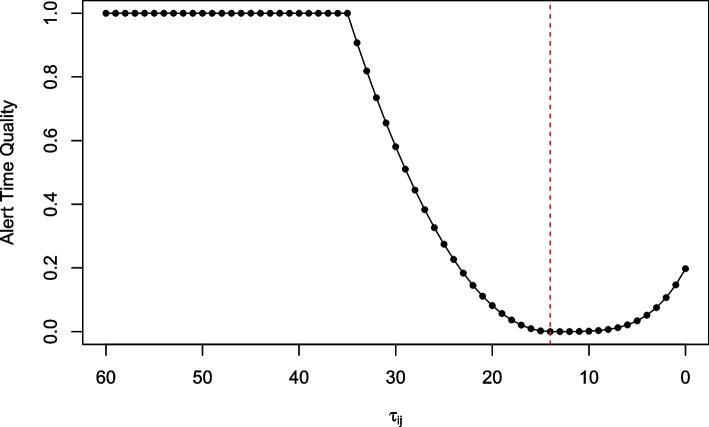
Alert time quality values corresponding to the time than an alert was raised. Alert times are relative to the number of days prior to the reference date ($\tau _{ij}=0$)

The proposed ATQ was developed based on the following principles that are relevant specifically to an influenza epidemic: (1) an optimal alert is raised 14 days prior to the reference date, (2) it is preferred that an alert is raised during the time interval of one to two weeks prior to the reference date, and (3) alerts raised marginally before or after the optimal alert are informative but their timeliness should be penalized in comparison to the optimal alert. The ATQ for alert *i* raised in year *j* was therefore defined as:

3 $$\begin{aligned} ATQ_{ij} = \left\{ \begin{array}{ll} \left( \frac{\tau _{opt} - \tau _{ij}}{k\tau _{opt}}\right) ^{2a} &{} \text {if } \tau _{ij} \le \tau _{opt} \\ \left( \frac{\tau _{opt} - \tau _{ij}}{k\tau _{opt}}\right) ^a &{} \text {if } \tau _{opt} < \tau _{ij} \le (k+1)\tau _{opt} \\ 1 &{} \text {if } \tau _{ij} > (k+1)\tau _{opt} \\ \end{array}\right. , \end{aligned}$$

where $\tau _{ij}$ was the number of calendar days before the reference date that the alert was raised. In lieu of strict true and false alert definitions, a power function conditional on when an alert was raised in relation to the optimal alert day ($\tau _{opt}$) was used to penalize alerts raised prior to the optimal day more than equidistant alerts raised after the optimal day. Following suggestions from WDGPH, an alert raised two weeks (14 days) prior to the reference date is considered to be optimal, as this allows sufficient time for WDGPH to implement intervention strategies to mitigate the spread of disease. Therefore $\tau _{opt}$ was set to 14. In addition, the power parameter *a* was set to 2, as it gave a reasonable increase of the penalty applied to alerts that are raised within a few days before and after $\tau _{opt}$ which, in discussion with WDGPH, were still deemed to be useful alerts. Since the reference date was different every year, the maximum possible value of the numerator changed every year. Thus, the ATQ was restricted to be a value between 0 and 1 by normalizing on a period from the optimal alert day, and the period must be reasonably wide to cover the difference between the $\tau _{opt}$ and the $\tau _{ij}$. For this study, the value of $k=1.5$ was suggested, resulting in a 21 day normalizing period. Consequently, alerts raised in the 21 days prior to $\tau _{opt}$, which may still be useful to a public health unit, generated a (penalized) ATQ value. Alerts raised prior to the $(k+1)\tau _{opt} = 2.5\tau _{opt}$ period from the optimal alert were considered too early for public health officials to effectively implement mitigation strategies, and were assigned the maximum value of 1. The ATQ yielded its minimum value of 0 when an alert was raised exactly on the optimal day, and increased as an alert was raised earlier or later than the optimal day. Visually, the ATQ embodied these principles by creating a valley shaped curve to assess an alert (Fig. 1). Additional details of the ATQ are provided in the supplementary information (see Supplementary Information).

### Model evaluation

The ATQ assesses the quality of a single alert raised. In order to evaluate how a statistical model performs in general, a model evaluation metric should take into account all alerts raised over one or several years. Here, we propose the following evaluation metrics based on the summary statistics of ATQs over all raised alerts: average alert time quality (AATQ), first alert time quality (FATQ), and their weighted counter parts. Each evaluation metric is also used as model parameter selection criterion, to select model lag, *l*, and threshold parameter $\theta ^*$. A brief algorithm for implementing an ATQ-based approach is provided in the supplementary information (see Supplementary Information).

#### Average alert time quality

The AATQ is defined as the yearly average ATQ value, averaged over all *J* years under consideration. To account for cases where there is no alert raised for some years, the AATQ is calculated as:

4 $$\begin{aligned} AATQ = \frac{\sum \limits _{j=1}^{J} AATQ_j}{J} , \end{aligned}$$

where *J* is the number of years evaluated and

5 $$\begin{aligned} AATQ_j = \left\{ \begin{array}{ll} \frac{\sum \limits _{i=1}^{n_j} ATQ_{ij}}{n_j} &{} \text {if an alert is raised in school year } j\\ 1 &{} \text {if no alerts are raised in school year } j, \\ \end{array}\right. \end{aligned}$$

with $ATQ_{ij}$ being the ATQ (Eq. 3) for the *i*th alert raised in school year *j*, and $n_j$ is the number of alerts raised in school year *j*.

#### First alert time quality

In practice, public health units would likely react and implement behavioural intervention strategies when the first alert is raised, therefore the quality of the first alert raised in a given year is crucial. The FATQ evaluates only the first alert raised in each year, with subsequent alerts not affecting the FATQ value. The FATQ is calculated as:

6 $$\begin{aligned} FATQ = \frac{\sum \limits _{j=1}^{J} FATQ_j}{J}, \end{aligned}$$

where *J* is the number of years evaluated and

7 $$\begin{aligned} FATQ_j = \left\{ \begin{array}{ll} ATQ_{1j} &{} \text {if an alert is raised in school year } j\\ 1 &{} \text {if no alerts are raised in school year } j\\ \end{array}\right. \end{aligned}$$

with $ATQ_{1j}$ being the ATQ (Eq. 3) of the first alert raised in year *j*.

#### Weighted AATQ and FATQ

The epidemic prediction model for each year is trained using data from all its preceding years, thus prediction models fit for later school years have more data available than models fit for earlier school years. In the computation of the AATQ and FATQ, alerts raised in all prediction years are weighted equally. However the epidemic prediction model in later school years have more data and because of this, could potentially fit the data better and have better predictions than models fit for earlier school years. To account for this, weighted versions of the AATQ and FATQ were developed. The weight applied to each years’ prediction is calculated based on the number of years used in its training set, divided by the total number of years to be predicted. For example, the weight applied to year *j*’s prediction is defined as:

8 $$\begin{aligned} w_j = \frac{\text {Number of years in epidemic prediction model for year } j}{\sum _{i=1}^n \text {Number of years in epidemic prediction model for year } i}, \end{aligned}$$

where *n* is the total number of prediction years in the study. This weight is used to compute the weighted average alert time quality (WAATQ) and weighted first alert time quality (WFATQ) metrics, given by:

9 $$\begin{aligned} WAATQ = \sum _{j=1}^J w_j AATQ_j \end{aligned}$$

and

10 $$\begin{aligned} WFATQ = \sum _{j=1}^J w_j FATQ_j \text { .} \end{aligned}$$

The model that produces the smallest value of the evaluation metrics is selected as the preferred model. The quality of alerts raised by models selected using AATQ, FATQ, and their weighted counterparts are compared graphically to the quality of alerts raised by the model selected using the FAR metric proposed by Ward *et al.* (2019) [10]. Additionally, to summarize the timeliness of alerts raised over all prediction years, alerts are categorized as follows: too late (alert raised 0-3 days prior to the reference date), slightly late (alert raised 4-6 days prior to the reference date), ideal (alert raised 7-14 days prior to the reference date), slightly early (alert raised 15-21 days prior to the reference date), and too early (alert raised more than 21 days prior to the reference date). For the purposes of this analysis, an acceptable alert is defined as an alert raised between 4-21 days prior to the reference date, encompassing alerts that are categorized as slightly early, ideal, and slightly late. Alerts raised in this range provide sufficient time for public health officials to implement mitigation strategies prior to the start of the epidemic, while not being so early in the season that residents may not feel cause to follow recommendations.

### Simulation study

Although the performance of proposed metrics can be assessed using the observed yearly data, a more intensive simulation study is conducted to thoroughly compare the quality of alerts raised by the proposed ATQ-selected models to that of the FAR-selected models. In order to generate a school absenteeism-based surveillance system, we developed a simulation model that consists of three sequential parts: 1) a population of individuals is generated; 2) annual influenza epidemics are simulated over the population; 3) probabilistic models are applied to the population and epidemic to generate school absenteeism and laboratory-confirmed influenza case data. The following sections detail each part of the data simulation.

#### Population simulation

An 80 x 80 square is used to represent a given study region, in this case the WDG region in Ontario, Canada. The region is divided into 16 equally sized square subregions, mimicking school catchment areas, to allow for heterogeneous population demographic characteristics across the region. For each subregion the number of elementary schools and the school size are simulated using Gamma($\alpha , \beta$) distributions. The $\alpha$ and $\beta$ parameters are estimated by fitting gamma distributions to the number of schools within each catchment area ($\hat{\alpha } = 4.313, \hat{\beta } = 3.027$) and the school sizes using the absenteeism data set provided by WDGPH ($\hat{\alpha } = 5.274, \hat{\beta } = 0.014$). The number of elementary schools within each subregion is drawn from the resultant Gamma(4.313, 3.027) distribution and rounded to the nearest integer. Similarly, the size of each school is drawn from the resultant Gamma(5.274, 0.014) distribution and rounded to the nearest integer.

The generated population consists of two subpopulations: (1) households with children, and (2) households without children. Household structures are generated for both subpopulations using probabilistic models based on available demographic information from WDG’s 2016 Census profile [17]. It is assumed that a household has a maximum of five people; within those five people, there is a maximum of three children. Subpopulation 1 is generated according to the distributions of lone or coupled parents, number of children by parent type, and age categories approximated by the WDG Census profile [17]. Each household with children would generate a parent type, and the number of children. However, since not all children within a household are elementary school aged, an approximation based on the census age categories is used to generate elementary school aged children [17]. Therefore the probability of a simulated child within a household attending an elementary school was assumed to be the proportion of the population under 20 years old (child age) that is aged 5 -14 years old (elementary school age). For each school, households with children would be generated until the schools’ size was achieved. Households with children were assigned to the schools’ corresponding subregion. However, only elementary school aged children are included in the absenteeism data. For example, if an elementary school in a subregion had a simulated size of 300, households with children would be generated and assigned to that school until there were 300 children of elementary school age generated.

After subpopulation 1 is generated, subpopulation 2 is then generated based on the distribution of household size and the proportion of households without children from the WDG Census profile [17]. For each subregion, the proportion of households without children is used to determine how many additional households without children are generated. For example, if the proportion of households without children is 50%, and a catchment area had 500 households with children, then 500 additional households without children are generated for that subregion. For each of these households, a probabilistic model is used to simulate the number of household members. Locations of each household was generated by complete spatial randomness within the subregions’ 20 x 20 boundaries. In total, 16 subregion populations are generated. Due to the computational cost associated with epidemic simulation, approximately a quarter of the WDG population, which is about 85,000 individuals representing approximately 34,000 households, is simulated for illustrative purposes.

#### Epidemic simulation

Individual level models (ILMs), as outlined in Deardon *et al.* (2010) [18], have been used to model and simulate epidemic data. ILMs allow for individual level effects and can be used for modelling spatial or contact-based infections. In our simulation study, a homogeneous spatial ILM is used to simulate epidemics in a susceptible, infectious, and removed (SIR) framework, using the epidata function in the EpiILM R package [19]. Model parameter values are selected based on the typical spread of influenza in the WDGPH region, and selected such that the resulting infection achieves its peak number of daily new infections within a few days of the start of the epidemic, and then decreases to no new daily infections over a few weeks, with approximately 3-11% of the population becoming infected each year [20]. The infectious period is set to 4 days [21]. Epidemic curves are visually inspected to determine if an influenza epidemic grew and decayed reasonably, and unreasonable epidemics are discarded. In total, 100 epidemics (10 years/replicated with 10 replications) are simulated for our simulation study. Additional information on the ILM parameters and their values are in the supplemental information (see Supplemental Information).

Without loss of generality, we set September 1 to be day 1 ($t=1$) of a given school year. The date the epidemic is initiated is generated from a $N(45, 15^2)$ distribution. A mean of 45 days after September 1, which is October 16, is used because this day is close to the mean occurrence day of the first influenza case observed in the WDGPH data. Generated start times of less than 20 days are reassigned to 20 days, since in a normal year the influenza epidemic is unlikely to begin that early within a school year. Epidemics are initiated by randomly infecting two individuals from each of the 16 subregions (32 individuals in total), with a random infection time that is within 14 days after the epidemic is initiated. The maximum epidemic length is set to $t=270$ days to resemble the seasonal influenza epidemic ending by the end of May, however since we are primarily interested in the start of the epidemic the length of the epidemic is not critical in this study.

#### Laboratory case confirmation and absenteeism system

Laboratory-confirmed influenza and absenteeism data sets are generated based on probabilistic models, because not all infected individuals seek medical attention and not all student absences are due to illness. The true proportion of individuals infected with influenza each year is unknown within the WDG population, and thus, so is the percentage of infected individuals that receive laboratory confirmation. Therefore, the laboratory confirmation rate is estimated as follows. We first estimate the number of individuals within WDG that would be infected with influenza using the WDG census population size multiplied by the conservative assumption of 3% of the population is infected with influenza each year [17, 20]. Then, the mean number of laboratory confirmed cases reported each year from the influenza data set provided by WDGPH is divided by the estimated number of infected individuals in WDG to obtain the mean proportion of infected individuals that receive laboratory confirmation in the region. The resultant estimate is about 2% of infected individuals receive influenza laboratory confirmation each year. The 2% laboratory confirmation rate is spread equally across the 4 day infection period, resulting in a 0.5% daily probability that an infected individual receives laboratory confirmation during their infectious period. Simulated laboratory-confirmed influenza cases counts are aggregated daily, and a binary variable is generated to indicate if at least one laboratory-confirmed case occurred on a given day. Reference dates for each epidemic are calculated as described in the Influenza data set section.

In this study, we considered two simplified scenarios for a child to be absent from school: (1) a child did not have influenza and was absent, or (2) a child had influenza and was absent. Here, Scenario 1 was considered as the baseline absenteeism, comprised of all other causes of absences. Since students are unlikely to be absent due to influenza illness early in the school year, a baseline absenteeism of 5% per day was estimated using absenteeism data provided by WDGPH from all September months. When a student was infected by influenza, it was assumed that there was a 95% chance they were absent from school each day until recovered. Based on both sources of absenteeism, daily all-cause absenteeism percentage was calculated for each school. Mean all-cause school absenteeism percentage was calculated daily to generate absenteeism data sets, as described in Absenteeism data set section.

For the purposes of illustrating the performance of the proposed evaluation metric model, the epidemic is simulated using a simplified model. Thus, statistical models without school year random effects and DOW indicators are considered for raising alerts in the simulated data set. A logistic regression model with lagged absenteeism and fixed seasonal terms given by:

11 $$\begin{aligned} \text {logit}(\theta _{tj}) =&\beta _0 + \beta _1 x_{tj} + \beta _2 x_{(t-1)j} + ... + \beta _{l+1} x_{(t-l)j}\nonumber \\&+ \beta _{l+2} \sin _j(\frac{2\pi t^*}{T^*}) + \beta _{l+3} \cos _j(\frac{2\pi t^*}{T^*}), \end{aligned}$$

is used to fit the simulated data. No random effect is included in the above model, as data are generated independently from year to year, and we assume a closed population such that no infections are from outside of the population as the epidemic progresses. All covariates are as described in the Epidemic detection models section.

A total of ten replications, each consisting of ten annual epidemics, is simulated. For each replication, logistic regression models (Eq. 11) are fit to the simulated data of each year, and lag and threshold parameters are selected for each replication using the proposed evaluation metrics. Quality of the alerts raised by ATQ and FAR-selected models are assessed across the ten replications.

## Results

### Preliminary data analysis of WDGPH data

**Fig. 2:**
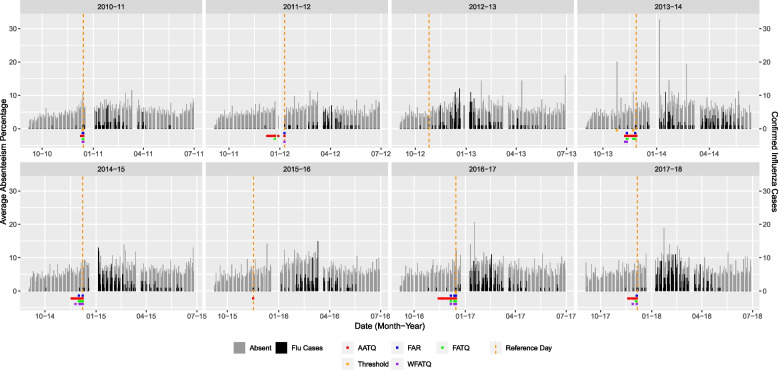
Daily average elementary school absenteeism and laboratory-confirmed influenza cases in WDG based on WDGPH data with alerts raised by selected models. Each panel represents a school year. Daily average absenteeism is plotted as grey bars, laboratory-confirmed influenza case counts are overlaid with black bars, and the epidemic reference day is indicated by the dashed orange lines. Coloured squares below the X-axis represent the date an alert was raised by each of the selected models

Daily average school absenteeism over 88 elementary schools and daily counts of the laboratory-confirmed influenza cases for WDG over the study period are shown in Fig. 2. Among 88 elementary schools, on a given day there were between 0 to 40 schools reporting absenteeism to WDGPH, with a median of 10 schools. Throughout the study period, only nine schools reported absenteeism on more than 50% of the available school days. In total, 1,697 out of 1,746 school days had recorded absenteeism data in the study period, based on an assumed 194 day school year. There was a mean daily all-cause absenteeism of 5.94% year round, whereas for all September months the mean was 5.29%. The Spearman correlation between laboratory-confirmed influenza case counts and mean school absenteeism was 0.371 [10], and cross-correlation was highest when there was a six day lag between school absenteeism and laboratory-confirmed influenza case counts (0.405) [10]. Across all study years, epidemic reference dates ranged from late October to late January, and occurred most frequently in December (Table 1).

**Table 1:** Reference dates for each year of the WDGPH data, representing the beginning of each seasonal influenza epidemic. A reference date is defined as the date of the second laboratory-confirmed influenza case within a seven day period for the first time within an influenza season

School Year	Reference Date
2008-09	January 20, 2009
2010-11	December 14, 2010
2011-12	January 9, 2012
2012-13	October 26, 2012
2013-14	November 27, 2013
2014-15	December 8, 2014
2015-16	November 17, 2015
2016-17	December 15, 2016
2017-18	December 6, 2017

### Epidemic alerts assessment for WDGPH data

**Fig. 3:**
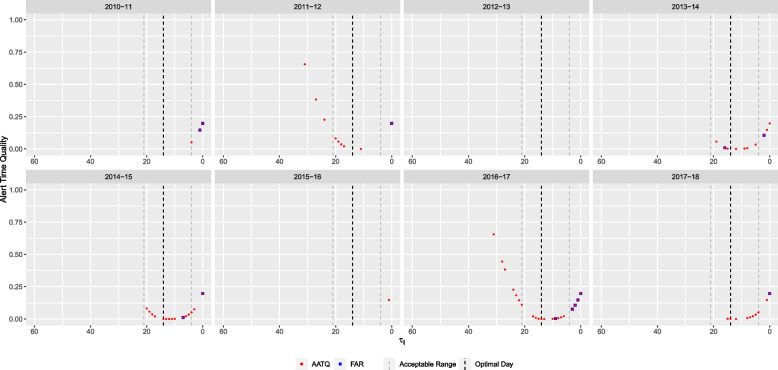
Alert time comparison between the AATQ and FAR-selected models based on WDGPH data. Alert times are relative to the reference date ($\tau =0$). Each school year is represented in its own panel. The vertical black dashed line represents the optimal alert time ($\tau = 14$). The vertical grey dashed lines represent the boundaries for acceptable alerts ($\tau = 21$ and $\tau = 4$)

**Fig. 4:**
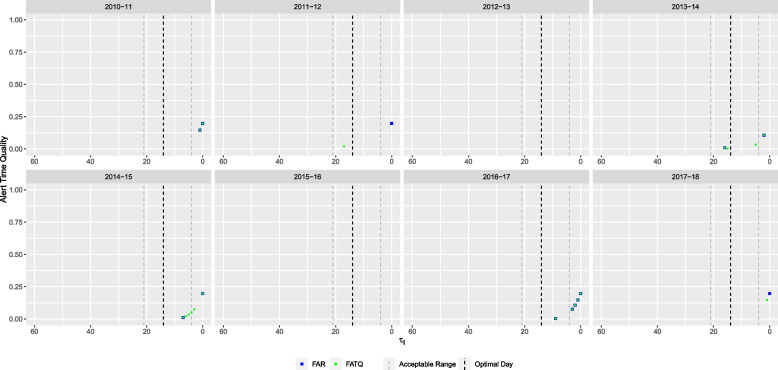
Alert time comparison between the FATQ and FAR-selected models based on WDGPH data. Alert times are relative to the reference date ($\tau =0$). Each school year is represented in its own panel. The vertical black dashed line represents the optimal alert time ($\tau = 14$). The vertical grey dashed lines represent the boundaries for acceptable alerts ($\tau = 21$ and $\tau = 4$)

Based on the FAR, the seasonal mixed model with lag time $l=11$ and threshold $\theta ^* =0.25$ (Table 2) was selected. Figure 2 shows the timing of alerts raised by this FAR-selected model, as well as all selected ATQ-based models, relative to daily absenteeism and laboratory-confirmed influenza cases in WDG. The FAR-selected model raised alerts for six out of eight years (Figs. 3 and 4). For three years (2013-14, 2014-15, and 2016-17), alerts were raised within the acceptable range, and for the remaining three years (2010-11, 2011-12, and 2017-18), alerts were raised too late by the FAR-selected model.

**Table 2:** Models selected by each metric based on the WDGPH data, and its lag and threshold values. Bold values indicate the minimum value of the given metric

Model	Parameters	FAR	AATQ	FATQ	WAATQ	WFATQ
Seasonal Mixed	$l=11, \theta ^*=0.25$	**0.3125**	0.3545	0.3208	0.3512	0.3123
Seasonal Mixed	$l=15, \theta ^*=0.15$	0.6043	**0.2171**	0.3312	**0.1694**	0.2912
Seasonal Mixed	$l=15, \theta ^*=0.25$	0.4583	0.3186	**0.2923**	0.3224	0.2912
Seasonal Mixed, DOW	$l=11, \theta ^*=0.25$	0.3750	0.3351	0.3021	0.3227	**0.2747**

The seasonal mixed model with lag time $l=15$ and threshold $\theta ^*=0.15$ was selected by the AATQ and WAATQ (Table 2). This model raised alerts in seven out of eight years (Fig. 3), whereas in four years (2010-11, 2013-14, 2014-15, and 2017-18) the first alerts were raised within the acceptable range. The AATQ/WAATQ-selected model raised more timely first alerts than the FAR-selected model in 2010-11 and 2017-18, and raised an alert in 2015-16 when the FAR-selected model failed to do so. In 2013-14 and 2014-15, the FAR-selected model produced first alerts closer to the optimal alert day than the AATQ/WAATQ-selected model, however the AATQ/WAATQ-selected model still produced acceptable alerts within these years. There were two years in which the AATQ/WAATQ-selected model raised alerts too early (2011-12 and 2016-17), of which the FAR-selected model raised alerts within the ideal range in one year, but raised an alert too late in the other.

Using the FATQ, the selected model was the seasonal mixed model with lag $l=15$ and threshold $\theta ^* = 0.25$ (Table 2), a larger lag value than the FAR-selected model and a larger threshold value than the AATQ-selected model. This model raised alerts for six out of eight years (Fig. 4). In four of these years (2011-12, 2013-14, 2014-15, and 2016-17), the first alert was raised within the acceptable range. The FATQ-selected model raised first alerts with timing better than (2011-12 and 2017-18), or equal to (2010-11, 2013-14, 2014-15, and 2016-17) that of the FAR-selected model. In addition, the FATQ-selected model generally produced more alerts in these years, potentially providing more confidence to public health officials in predicting an imminent epidemic.

**Fig. 5:**
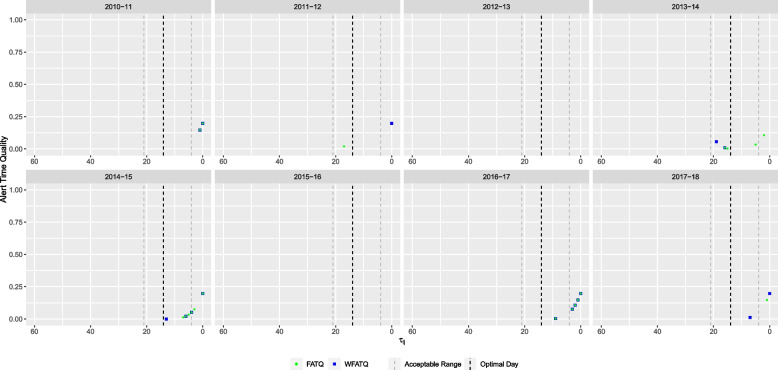
Alert time comparison between the FATQ and WFATQ-selected models based on WDGPH data. Alert times are relative to the reference date ($\tau =0$). Each school year is represented in its own panel. The vertical black dashed line represents the optimal alert time ($\tau = 14$). The vertical grey dashed lines represent the boundaries for acceptable alerts ($\tau = 21$ and $\tau = 4$)

When using the WFATQ metric, the selected model was the seasonal mixed DOW model with lag time $l=11$ and threshold $\theta ^* = 0.25$ (Table 2). The WFATQ-selected model raised alerts for six out of the eight years (Fig. 5). The WFATQ-selected model raised alerts with timing equal to or better than that of alerts raised by the FATQ-selected model in later years.

**Fig. 6:**
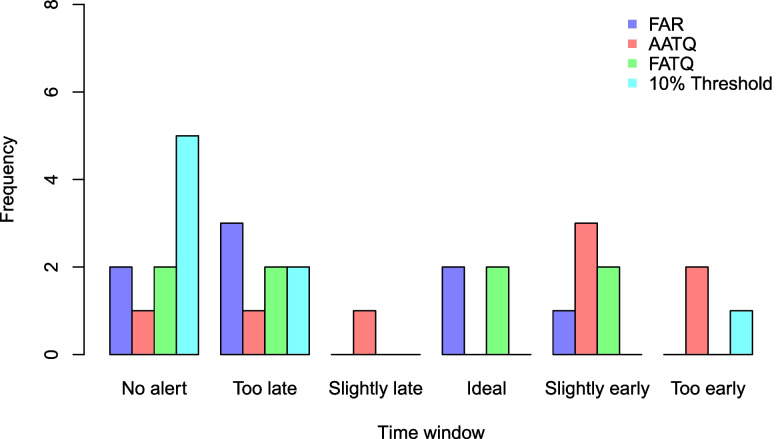
First alert categorization frequencies based on WDGPH data, under selected models. Alerts are categorized as follows: too late (alert raised 0-3 days prior to the reference date), slightly late (alert raised 4-6 days prior to the reference date), ideal (alert raised 7-14 days prior to the reference date), slightly early (alert raised 15-21 days prior to the reference date), and too early (alert raised more than 21 days prior to the reference date). An acceptable alert is an alert categorized as slightly late, ideal, or slightly early

Overall, the performance of a model-based approach using either ATQ-based or FAR evaluation metrics is much better than that of the current 10% threshold approach. As illustrated in Fig. 6, the 10% absenteeism threshold approach either failed to raise an alert or raised the first alert too late or too early. The AATQ and FATQ-selected models raised the most first alerts that were within the acceptable range, while the FAR and FATQ-selected models tied for the most first alerts raised within the ideal range throughout the eight years (Table 3). None of the selected models raised alerts for the 2012-13 school year. Alerts raised by the WAATQ and WFATQ-selected models were not included in Fig. 6, since by design they do not perform as well as the unweighted metrics for earlier school years but may outperform the unweighted metrics in the later school years.

**Table 3:** First alert categorization frequencies based on WDGPH data, under selected models

Optimized Metric	No Alert	Too late 0-3 days before ref.	Slightly late 4-6 days before ref.	Ideal 7-14 days before ref.	Slightly early 15-21 days before ref.	Too early 22+ days before ref.
10% Threshold	5	2	0	0	0	1
FAR	2	3	0	2	1	0
AATQ	1	1	1	0	3	2
FATQ	2	2	0	2	2	0
WAATQ	1	1	1	0	3	2
WFATQ	2	2	0	3	1	0

### Epidemic alerts assessment for the simulation study

**Fig. 7:**
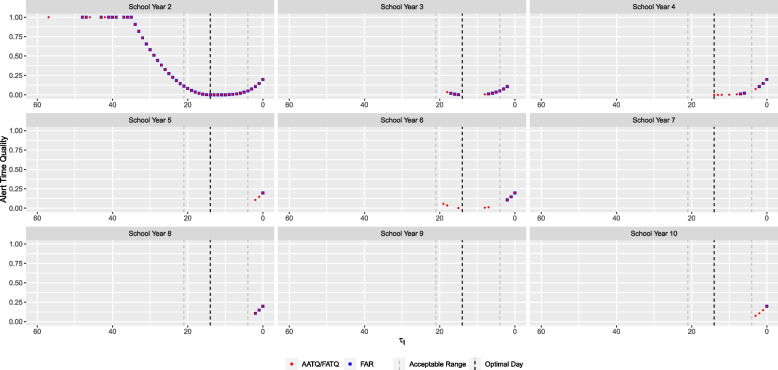
Alert time comparison between the AATQ and FAR-selected models based on one simulated replication. Alert times are relative to the reference date ($\tau =0$). Each school year is represented in its own panel. The vertical black dashed line represents the optimal alert time ($\tau = 14$). The vertical grey dashed lines represent the boundaries for acceptable alerts ($\tau = 21$ and $\tau = 4$)

**Fig. 8:**
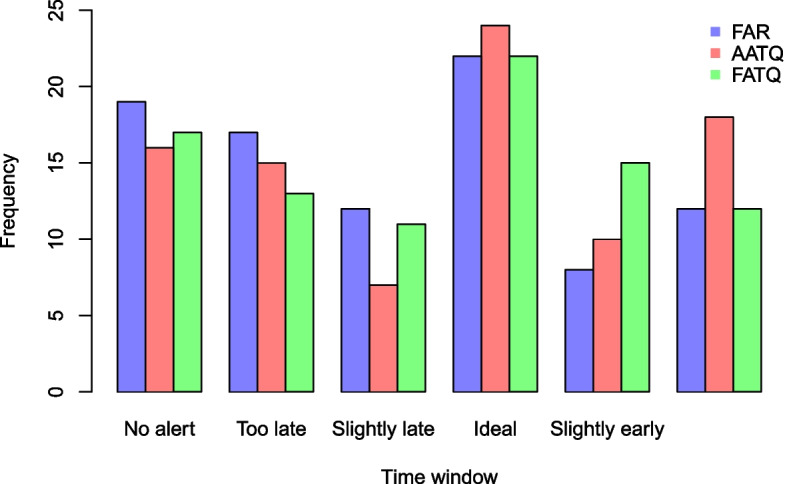
First alert categorization frequencies based on simulated data, under selected models. Each of the 10 replications include 9 prediction years. Alerts are categorized as follows: too late (alert raised 0-3 days prior to the reference date), slightly late (alert raised 4-6 days prior to the reference date), ideal (alert raised 7-14 days prior to the reference date), slightly early (alert raised 15-21 days prior to the reference date), and too early (alert raised more than 21 days prior to the reference date). An acceptable alert is an alert categorized as slightly late, ideal, or slightly early

For illustration purposes, the ATQ of alerts raised by the AATQ and FAR-selected models for one simulated replication are displayed in Fig. 7. For this particular replication, the AATQ and FATQ metrics selected the same model lag and threshold parameters, however this was not consistent across replications. Table 4 and Fig. 8 summarize the frequency of first alerts raised within each time window for models selected using ATQ-based and FAR evaluation metrics over all replications. Overall trends of the simulation study showed that the first alert raised by AATQ and FATQ-selected models were often more timely than the first alert raised by the FAR-selected model. Additionally, the FAR-selected model failed to raise an alert or raised the first alert too late more frequently than all ATQ-selected models (Fig. 8, Table 4). The unweighted ATQ-based and FAR-selected models raised their first alert within the ideal range more frequently than in any other time category. Using the conventional 10% absenteeism threshold method, no alerts were raised prior to the first simulated influenza case for any of the replications. Overall, the FATQ-selected model had the highest proportion of first alerts raised within the acceptable time range (53%). The proportion of first alerts raised within the acceptable time range was comparable between the AATQ and FAR-selected models, at 45% and 47% respectively. However, the AATQ-selected model had the most first alerts raised within the ideal time range (27%), but also raised first alerts too early more frequently than the models selected by FAR and FATQ. Generally, no alerts were raised, or the first alert was raised outside of the acceptable time range, in school year 2, evidenced by higher mean and median metric values than those in later school years regardless of the model selection criterion used (Table 5).

**Table 4:** First alert categorization proportions based on simulated data, under selected models over 10 replications of 9 prediction years

Model	No alert	Too late 0-3 days before ref.	Slightly late 4-6 days before ref.	Ideal 7-14 days before ref.	Slightly early 15-21 days before ref.	Too early 22+ days before ref.
10% Threshold	1.00	0.00	0.00	0.00	0.00	0.00
FAR	0.21	0.19	0.13	0.24	0.09	0.13
AATQ	0.18	0.17	0.08	0.27	0.11	0.20
FATQ	0.19	0.14	0.12	0.24	0.17	0.13
WAATQ	0.18	0.16	0.11	0.20	0.13	0.22
WFATQ	0.20	0.18	0.13	0.19	0.13	0.17

**Table 5:** Evaluation metric measures of variability for each year of the simulated data, under selected models over 10 replications of 9 prediction years

Model	Measure	Year 2	Year 3	Year 4	Year 5	Year 6	Year 7	Year 8	Year 9	Year 10
FAR$_j$	Mean	0.6973	0.2250	0.2300	0.5875	0.3625	0.3911	0.2500	0.3500	0.3395
	Variance	0.1590	0.1451	0.1512	0.2562	0.2276	0.2552	0.1806	0.2250	0.2104
AATQ$_j$	Mean	0.4218	0.1656	0.1707	0.3894	0.2898	0.2250	0.2671	0.3649	0.1335
	Variance	0.1740	0.0890	0.0876	0.1793	0.1466	0.0801	0.1522	0.1933	0.0115
WAATQ$_j$	Mean	0.4310	0.3623	0.1761	0.3996	0.1995	0.3058	0.0780	0.3809	0.1254
	Variance	0.1790	0.1959	0.0867	0.1729	0.0857	0.1404	0.0032	0.1839	0.0134
FATQ$_j$	Mean	0.5699	0.2607	0.1428	0.5086	0.3322	0.3375	0.1510	0.3340	0.2749
	Variance	0.2207	0.1760	0.0946	0.2211	0.2141	0.2127	0.0939	0.2134	0.1576
WFATQ$_j$	Mean	0.5852	0.4114	0.3669	0.4679	0.2046	0.2583	0.1886	0.3534	0.1760
	Variance	0.2035	0.2257	0.2216	0.1851	0.1166	0.1570	0.0891	0.2029	0.0981

Comparison between the weighted and unweighted ATQ-selected models is not suggested for overall years since both the weighted and unweight method do not perform well for earlier school years due to smaller sample sizes in training the prediction models. Instead, the quality of alerts raised by the weighted and unweighted ATQ-based methods for the last five years of each replication are compared and summarized in Table 5. The WAATQ-selected model had a lower mean and variance of AATQ$_j$ value in three of the last five years of the simulation study when compared to the unweighted AATQ-selected model. The WFATQ-selected model had a lower mean FATQ$_j$ in three of the last five years and the variances were consistently smaller for those selected by WFATQ models. This shows that models selected by weighted metrics generally outperform their unweighted counterparts in later years.

## Discussion

In this study, we proposed a novel metric, ATQ, to simultaneously evaluate the accuracy and timeliness of alerts raised by a school absenteeism-based syndromic surveillance system. With the proposed ATQ for each raised alert, the summary statistics of the ATQ such as AATQ and FATQ were proposed to be used as model selection criteria when historical data (previous years’ data) are available for training a prediction model to raise an alert. These metrics were shown to be useful and practical for selecting a model that raised an alert for an approaching seasonal influenza epidemic in a timely manner, and were shown to generally select a model that raised more quality alerts than the FAR-selected model or the 10% absenteeism threshold method. A simulation study was conducted with two primary objectives: to investigate the performance of the proposed metrics in a simulated data set, which was not subject to the inherent uncertainties commonly found in observed data, and to develop an infectious disease/school absenteeism simulation framework which can be adopted for simulation studies associated with other infectious diseases and regions.

Measures such as sensitivity and specificity have commonly been used to evaluate the performance of surveillance systems [22]. These measures are usually combined into a single metric characterized by the area under the receiver operating characteristic (ROC) curve, to be used for comparisons across models. However, these measures do not capture the timeliness of an alert, which is crucial for surveillance [22]. Kleinman and Abrams extended the ROC curve to include timeliness [23], but this extension defines a true alert as the one that overlaps with an epidemic, and measures time between the beginning of the epidemic and the first alert. That means their defined true alert will not occur before the epidemic start and therefore loses the sense of being an early warning alert. In contrast, our proposed ATQ would help to train a more precise epidemic detection model using previous years’ reported influenza cases and school absenteeism data for raising an alert prior to when the epidemic would be declared. This approach provides a practical solution to public health agencies for laying out timely interventions in order to slow the epidemic or reduce the number of infections. To the best of our knowledge, there are no other metrics available yet that can evaluate the accuracy and timeliness of alerts simultaneously, and prior to the start of an epidemic.

While the ATQ-based metrics outlined in this study are successful in selecting an epidemic detection model that raises higher quality alerts than the FAR-selected model, there are limitations. Since the AATQ averages all alerts within a year, in the extreme case, for example, when an alert is raised everyday until the epidemic reference date in a given year, the AATQ would produce a value less than 1 (the maximum or worst value of AATQ) for that year despite the poor specificity of this model. The FATQ does not have the same limitation since its calculation uses the first alert raised in a given year, and therefore would give its maximum value of 1 to penalize the given model. In addition, the AATQ would favour an epidemic detection model that raises one alert at the optimal time over any other model, including an alternative case that a model raises an alert every day during the ideal range. In the case that the model raises an alert every day within the ideal range, these alerts intuitively provide more confidence to public health agencies to implement interventions. However, in terms of quality assessment, raising multiple alerts results in non-zero ATQ for those not raised on the optimal day and thus increases the AATQ. With this limitation, the AATQ would favour the model that produced one single alert at the optimal time. However based on our study results, subsequent alerts often follow shortly after the initial alert and multiple alerts raised after the optimal time would increase the AATQ no matter what.

When analyzing the WDGPH data, no selected models were successful in raising an alert for the 2012-13 school year. Of all the years included in our data, the 2012-13 school year had the earliest epidemic reference date (October 26, 2012; Table 1), and there were only two preceding school years’ data available to train the detection model for that year. It is not surprising that the trained model failed to detect the incoming epidemic when the epidemic started unusually early, and there was limited training data. Similar results were found in the simulation study, where the models trained by only the first year and the September of the second year’s data failed to detect the start of the epidemic or raised poor quality alerts in the second year. The quality of alerts was improved for later years in which there were more preceding school years’ data included for training the detection models.

The quality of absenteeism data also plays an important role in training models for raising high quality alerts. The absenteeism data provided by WDGPH relied on the voluntary completion of the online forms filled by schools in the UGDSB that serves the WDG region. Only 5 out of 88 participating elementary schools consistently reported absenteeism information over the entire study period, leaving a substantial amount of missing data. Since daily absenteeism was averaged across all reporting schools, whether or not an alert would be generated would depend on the location of the reporting schools and might not be reflective of the whole region. For example, if initial influenza infected cases occurred in Wellington county but only schools within Guelph provided absenteeism data, the resulting alert might be raised too late for the whole WDG region. To enable reliable school absenteeism-based surveillance, it is important for all or most area schools to consistently report daily absenteeism data. Reducing the amount of missing data would improve the model fitting and possibly allow for a more accurate selection of an epidemic detection model for the entire region. This is particularly true for characterizing the baseline absenteeism in the trained models, such that the trained model would have a higher sensitivity to detect unusual absenteeism patterns due to influenza illness. Our simulation study was based on a scenario with no missing data, more closely mimicking a system with mandatory absenteeism reporting, although in reality, school absenteeism can only be collected on school days and thus will always be missing weekends and holidays. The difference in data quality, combined with an additional year of data and replications of a ten year study period, resulted in a more consistently better performance of the ATQ-based metrics over the FAR approach. This emphasizes the need for good quality absenteeism and influenza data over multiple years for training a model that raises better quality alerts using the ATQ-based metrics. However despite this discrepancy, both the WDGPH study and the simulation study led to similar results in general.

WDG is a predominantly rural region, with the exception being the City of Guelph, a relatively small area of WDG where approximately half of the region’s total population is located [17, 24]. Due to the wide spatial dispersion of the population, and of schools, in the rural areas, alerts raised by the influenza surveillance system may only pertain to a specific area of the greater region. Thus, if spatial parameters are incorporated into the epidemic detection models, localized rather than generalized alerts can be raised. While spatial locations of reporting schools were not provided for confidentiality reasons, school catchment area identifiers could be obtained without loss of anonymity, and might provide a suitable spatial approximation for localized epidemic detection improvement. However, in this study there was insufficient data to study catchment area alerts due to the small number of consistently reporting schools. Future work could incorporate catchment areas into the modelling when more complete data are available. Furthermore, with those data, our simulation model could be modified to generate data that incorporates catchment area information to evaluate the performance of selected models in terms of raising localized alerts. Finally, the use of a seasonal logistic regression model does not directly account for any spatial or spatiotemporal dependence between disease cases. The year-varying random effect included in this model will, to some extent, indirectly account for infections from outside the region or other unknown sources of variability. However, incorporating a more specific spatiotemporal component into the seasonal logistic regression model to directly account for these effects should be investigated in future work.

The simulated absenteeism and influenza laboratory confirmation system might not have been able to fully capture the dynamics of a real influenza epidemic. For example, studies have shown that students are more likely to be absent on Mondays and Fridays, and that school absence episodes often last a single day [25]. Additionally, the level of influenza transmission varies between weekdays and weekends [26]. Thus, a more refined simulation procedure to account for this variation by days of week may help to generate more realistic absenteeism data. Finally, our simulation study was based on the collective estimates of population parameters, such as the absenteeism rate and the rate at which individuals seek medical attention, that were specific to the WDG population. As such, the results of this study are specific to the WDG region and the spread of influenza. Extending the approach proposed here to another public health unit and/or a different infectious disease may result in different preferred metrics on which to optimize the proposed model, appropriate to a particular health unit’s objectives or the corresponding disease dynamics. Our simulation model can be applicable to generate data that are suitable for other regions or diseases by using estimates of parameters from the respective regions or disease components. Future work could include a thorough sensitivity analysis of the proposed metrics using simulation studies based on populations from different public health units with different population characteristics, or different infectious diseases with varying disease dynamics.

Based on the results of the analysis of both the real data and the simulated data in this study, we believe that the proposed ATQ metric is suitable to evaluate accuracy and timeliness of an alert raised by a given school absenteeism surveillance system for influenza and other respiratory virus activity. For the WDG region, we recommend the selection of a model that minimizes the FATQ. This approach raised the first alert within the acceptable time range most frequently in both the real data and simulation study. It is also suggested that the selected model, and its parameters, be updated annually to incorporate yearly influenza and school absenteeism data. In addition, our proposed ATQ metric can be modified to be used for other seasonal infectious diseases such as COVID-19, if that disease ultimately becomes seasonal, or it can be adapted for use in a different public health region.

## Supplementary Information

**Additional file 1.**

## Data Availability

Under a data sharing agreement, the data for this study were made available by, and used with permission from, Wellington-Dufferin-Guelph Public Health. Complete R code for the simulation study is publicly available (https://github.com/vanderkk/School_Abstenteeism_Based_Influenza_Surveillance_Simulation_Study).

## Abbreviations

AATQ: Average alert time quality
ADD: Accumulated days delay
ATQ: Alert time quality
DOW: Day of the week
FAR: False alert rate
FATQ: First alert time quality
UGDSB: Upper Grand District School Board
WAATQ: Weighted average alert time quality
WDG: Wellington-Dufferin-Guelph
WDGPH: Wellington-Dufferin-Guelph Public Health
WFATQ: Weighted first alert time quality
