# Supplementary material for: ATQ: alert time quality, an evaluation metric for assessing timely epidemic detection models within a school absenteeism-based surveillance system
Source: BMC Public Health. 2023 May 10;23:850. doi: 10.1186/s12889-023-15747-z (PMC10170459; doi:10.1186/s12889-023-15747-z)
Supplement: Supplementary file 1 — Additional file 1. [file 12889_2023_15747_MOESM1_ESM.pdf]

Alert Time Quality: Supplementary Information

Supplementary information 1 — ATQ details

Additional values for k and a in the ATQ were considered, in an attempt to identify the parameter values of the ATQ that provided adequate penalization for suboptimal influenza alerts. Values of $k =$ 1, 1.5, and 2 were examined, corresponding to 14, 21, and 28 days respectively given $\tau_{opt} = 14$. By inspection and consultation with WDGPH, we found that $k = 1$ penalized alerts that occurred between the reference date and a few days prior too heavily, while $k = 2$ did not penalize alerts raised several weeks prior to the optimal alert date enough. A balance was found with the choice of $k = 1.5$. Values of $a = 1$, 1.5, 2, and 2.5 were examined. Values of $a = 1$ and 1.5 were found to increase the ATQ value too rapidly both before and after the optimal alert date, which therefore penalized alerts within the acceptable alert range too severely, and did not provide the ideal gradual gradient for penalization. Conversely, when $a = 2.5$, the gradient produced was too slow, such that alerts that occurred a few weeks prior to the reference date were not sufficiently penalized, suggesting that these alerts are within the ideal alert range of 7 - 14 days prior to the reference date. The selected $a = 2$ value minimized the value of the ATQ during the ideal range and sufficiently penalized alerts raised outside of that range. When selecting parameters in consultation with WDGPH, the alert ranges of ideal, slightly too early/late, and too early/late were considered, such that the visual/numerical representation aligns with the intuition these ranges provide.

Supplementary information 2 — Epidemic Alert Raising Procedure

Suppose school absenteeism and influenza confirmed case data is available for J years. The prediction model for each given year is trained using data from the preceding year(s). Upon selecting one of the ATQ-based evaluation metrics (AATQ, FATQ, and their weighted counterparts) to be used, the following is a summary of the procedure for obtaining an optimal model to raise alert(s) for the incoming year.

1. For each given current year, $j = 2, ..., J$, the lag logistic regression model is trained on data from all preceding years and the month of September for the current year. For example, if $J = 4$ years of data are available, the lag logistic regression model for Year 2 is trained by using the data of Year 1 and September for Year 2; for Year 3 the model is trained by using the data of Years 1 and 2 and September for Year 3; and for Year 4 the model is trained by using the data of Years 1, 2, and 3, and September of Year 4.

2. For each current year j, use the trained lag logistic model to calculate θ_{tj}, the probability of at least one influenza case on day t in year j, based on the provided absenteeism data up to that day t.

3. Providing a combination of two sequences of parameters: a threshold value, θ, and the lag period, l, for each pair of (θ, l), an alert is raised on day t for the jth year if $\theta_{tj} > \theta$.

4. For each year j, compute the selected ATQ-metric to evaluate the quality of alerts raised for that year. The pair (θ, l) that minimize the ATQ-based metric becomes the optimal model parameters, denoted θ^* and l, to be used in the surveillance model.

Supplementary information 3 — ILM details

Under the ILM framework, the probability of a susceptible individual i becoming infected by an infectious individual in the time interval $[t, t+1)$, $P(i,t)$, was given by:

$$P(i,t) = 1 - \exp(-\alpha \sum_{j \in I(t)} d_{ij}^{-\beta}) \qquad \alpha, \beta > 0, i \in S(t), \tag{1}$$

where $S(t)$ and $I(t)$ denoted the set of all susceptible and infectious individuals at time t respectively; α was the infectivity parameter for contracting the disease; $d_{ij}^{-\beta}$ quantified the risk of infection of individual i depending on the distance from infectious individual j, with d_{ij} being the Euclidean distance between individual i and infectious individual j, and β was the spatial parameter for the geometric rate of decay. To simulate influenza epidemics in our population, the infectivity and spatial parameter were set as $\alpha = 0.0019$, and $\beta = 3$. Additionally, the infectious period was set to 4 days [21]. The parameter values of α and β were chosen such that the resulting epidemic reached its peak number of new infections within a few days of the start of the epidemic, but then the number of daily new infections decreased until there were no new daily infections a few weeks after the start of the epidemic. Epidemics that continued uniformly over a prolonged period, did not propagate at all, or began and ended within a few days are not typical of influenza, and therefore parameter values that resulted in epidemics of these types were not considered. The choice of parameter values depend on the population size, density, and the nature of the disease, thus the parameter values were chosen specifically for this study.
